# Plant-Derived Smoke Mitigates the Inhibitory Effects of the Auxin Inhibitor 2,3,5-Triiodo Benzoic Acid (TIBA) by Enhancing Root Architecture and Biochemical Parameters in Maize

**DOI:** 10.3390/plants12142604

**Published:** 2023-07-10

**Authors:** Gulfan Ullah, Muhammad Ibrahim, Ghazala Nawaz, Amana Khatoon, Muhammad Jamil, Shafiq Ur Rehman, Essam A. Ali, Akash Tariq

**Affiliations:** 1 Department of Botany, Kohat University of Science and Technology, Kohat 2600, Pakistan; gulfambetani88444@gmail.com (G.U.); bo320212003@kust.edu.pk (M.I.); amanakhatoon@kust.edu.pk (A.K.); jamilkhattak@yahoo.com (M.J.); 2 Department of Biology, The University of Haripur, Haripur 2262, Pakistan; 3 Department of Pharmaceutical Chemistry, College of Pharmacy, King Saud University, Riyadh 11451, Saudi Arabia; esali@ksu.edu.sa; 4 Xinjiang Key Laboratory of Desert Plant Roots Ecology and Vegetation Restoration, Xinjiang Institute of Ecology and Geography, Chinese Academy of Sciences, Urumqi 830011, China; akash.malik786@mails.ucas.ac.cn

**Keywords:** indole-3-acetic acid, indole-3-butyric acid, 2,3,5-triiodo benzoic acid, plant-derived smoke solution

## Abstract

The present study was designed to investigate and compare the effects of plant-derived smoke (PDS) and auxin (IAA and IBA) on maize growth under the application of 2,3,5-triiodo benzoic acid (TIBA). For this purpose, indole-3-acetic acid (IAA) and indole-3-butyric acid (IBA), each at a concentration of 10 ppm, along with PDS at a ratio of 1:500 (*v/v*) were used alone and in combination with 10 ppm of TIBA. The results indicate that the germination percentage (%) of maize seeds was enhanced under IAA, IBA and PDS treatment. However, IAA and IBA resulted in reduced germination when applied in combination with TIBA. Importantly, the germination percentage (%) was improved by PDS under TIBA treatment. The analysis of seedling height, length of leaves, and number of primary, seminal and secondary/lateral roots showed improvement under individual treatments of IAA and IBA, PDS and PDS + TIBA treatment, while these values were reduced under IAA + TIBA and IBA + TIBA application. Chlorophyll content, total soluble sugars and antioxidative enzymatic activity including POD and SOD increased in seedlings treated with PDS alone or both PDS and TIBA, while in seedlings treated with IAA and TIBA or IBA and TIBA, their levels were decreased. APX and CAT responded in the opposite way—under IAA, IBA and PDS treatment, their levels were found to be lower than the control (simple water treatment), while TIBA treatment with either IAA, IBA or PDS enhanced their levels as compared to the control. These results reveal that PDS has the potential to alleviate the inhibitory effects of TIBA. This study highlights the role of PDS in preventing TIBA from blocking the auxin entry sites.

## 1. Introduction

Fire is one of the most common ecological tools, and has the potential to play a significant role in vegetation growth all over the world [1]. Fire generates chemicals, ash, heat, and smoke, all of which have already been recognized as germination signals for the growth of a variety of species in fire-prone and fire-free habitats [2,3,4,5].

Plant-derived smoke (PDS) produced by the burning of plant parts such as leaves, shoots and stems accelerates the germination and growth process of over 1200 different plant species belonging to 80 different genera [6,7]. PDS, for the first time, was found to be involved in promoting seed germination and growth in plants of the South African Mediterranean [8,9] and Californian chaparral [10]. Moreover, it is widely proven that PDS breaks seed dormancy and accelerates plant growth [11,12,13]. Previously, PDS has been determined to contain active components that alleviate the effects of extremely harsh habitats on plants [14,15,16]. Reportedly, PDS has the ability to mitigate the effects of abiotic stresses such as temperature, salinity, heavy metals, and drought on plant growth [17]. PDS promotes seed germination and increases seedling length, root length, the number of leaves, leaf area, and root number in onion [18] and in banana [19]. Previously, PDS has been reported to exhibit hormone-like responses. It interacts with auxin, gibberellin, cytokinins, ABA, and ethylene in various plants [20]. Karrikins, such as 3-methyl-2H-furo [2,3-c] pyran-2-one, are a class of compounds consisting of butenolide fused to a pyran ring. They have been isolated from smoke and are active at very low concentrations up to 10^−10^ M, playing an important role in germination [20]. Recently, PDS has been determined to enhance the root system and positively affect physiological parameters including photosynthetic pigments and the level of antioxidant enzymes in chickpea [21], wheat [22], and maize [23].

Auxin is one of the primary hormones essential for the process of embryogenesis and seedling growth [24]. Auxin is primarily synthesized in young developing tissues like leaves and stems and is transported polarly throughout the plant [25]. Indole-3-acetic acid (IAA) and indole-3-butyric acid (IBA) are types of auxins that play significant roles in the growth and development of lateral and adventitious roots [26]. Previously, the application of IAA has significantly improved root growth at low concentrations, while inhibiting it at higher concentrations [27]. In addition, auxin is a key regulator of several developmental processes, including embryogenesis, the lateral branching of shoots and roots, cell elongation, tropic responses to light and gravity, and vascular tissue differentiation in plants [28].

2,3,5-triiodo benzoic acid (TIBA) is a well-known inhibitor of auxin (IAA and IBA) [29]. TIBA prevents polar auxin transport between cells and reduces auxin outflow [30]. It also competes directly with IAA at the efflux carrier and thus stops the transport of IAA in plants by occupying auxin transport channels [31].

Looking at the promoting effects and hormone-like responses of PDS, the present study was designed to evaluate the effect of PDS on root growth and the biochemical parameters of maize under TIBA application. This study will be the first initiative to compare the effects of PDS and auxin on maize under the treatment with TIBA. Therefore, it was hypothesized that PDS (1:500 *v/v*) may promote maize growth under the application of TIBA.

The present study aimed to evaluate and compare the effect of PDS, IAA and IBA alone and in combination with TIBA on morphological and biochemical parameters of maize seedlings. Our results demonstrate that compared to IAA and IBA, PDS significantly enhanced the germination percentage, morphological parameters such as plant height, root length, and number of primary, seminal and secondary roots, chlorophyll contents, level of TSS, carbohydrates, fiber contents and antioxidants enzymes such as SOD, POD, APX and CAT under TIBA treatment. This study suggests that the promoting effects of PDS were not altered on maize and PDS may play an important role in reducing the competition of TIBA for the auxin transport channels and could function in the regulation of internal auxin content, which needs further investigation.

## 2. Material and Methods

### 2.1. Seed Source, Selection, and Sterilization

Maize (*Zea mays* L.) seeds cv. Azam were obtained from the Agriculture Research Center, Sara-e-Naurang, Lakki Marwat (ARCSL), KP, Pakistan. Healthy and uniformly sized seeds were selected. Seeds were sterilized in a solution containing 1% sodium hypochlorite for five minutes, followed by washing with 70% ethanol for 3 min and rinsing with distilled water five times.

### 2.2. Preparation of Plant-Derived Smoke Solution

PDS solution was prepared from the leaves of *Cymbopogon jwarancusa* L., locally named “Sargarra”, by following the methods of Baxter and Van Staden [32]. Three hundred and thirty-three grams of semi-dried leaves were placed in a locally manufactured chimney. An electric heater with an element with a power of 2500 Watts was used to heat the chimney to burn the *C. jwancusa* leaves contained within it. Bubbles emitted from the burning of leaves were collected through a pipe in a volumetric flask containing 1000 mL of water. This solution was termed as stock solution [33]. The stock smoke solution was diluted to a 1:500 (*v/v*) concentration for further studies.

### 2.3. Preparation of IAA, IBA and 2,3,5-Triiodo Benzoic Acid (TIBA) Solutions

The IAA, IBA and TIBA were purchased from the international market of the United States of America (Sigma Aldrich, St Luis, Burlington, Massachusetts, USA). One molar (1 M) stock solutions of IAA, IBA and TIBA were prepared and diluted to 10 ppm for further experimental use.

### 2.4. Measurement of Germination Rate

The selected (healthy and uniformly sized) maize seeds were pre-soaked for 18 h in distilled water (control), IAA (10 ppm), IBA (10 ppm), PDS (1:500), IAA + TIBA (each 10 ppm), IBA + TIBA (each 10 ppm) and PDS + TIBA (1:500 + 10 ppm) before germination. The pre-soaked seeds were sown in 9 cm Petri plates lined with double layers of filter paper (Whatman No. 1). Seeds were supplied with 3-5 mL of each treatment solution, i.e., IAA, IBA, PDS, IAA + TIBA, IBA + TIBA and PDS + TIBA. In each experiment, 3 replicates were used, with 10 seeds per replicate. Seed germination was noted after the protruding of a radical and data were collected for 3 days. The whole experiment was conducted in a growth room which was maintained with a 14/10 h photoperiod, a light intensity of 500 µmol m^−2^ s^−1^, a day and night temperature of 24 and 19 °C, respectively, and 60–65% relative humidity.

### 2.5. Measurement of Morphological and Biochemical Parameters

After 10 days of seed germination, the plant height, length of leaves, number of seminal roots, number of secondary roots, and length of primary roots were measured carefully. Different physiological growth parameters including photosynthetic pigments (chlorophyll *a*, *b*, and total carotenoids) and total soluble sugars were measured.

### 2.6. Determination of Photosynthetic Pigments

To measure the photosynthetic pigments, fresh maize leaves (25 mg) were placed in a test tube and 10 mg of MgO and 5 mL of ethanol were added. After that, samples were homogenized in a shaker (Thermo Stable IS- 20R, DAIHAN Scientific Co., Ltd., Seoul, Republic of Korea) at 4000 rpm for 60 min. After centrifugation (D2012 plus, DLAB), the mixture was centrifuged at 4000 rpm for 5 min at room temperature, and the supernatants were obtained. The absorbance value was recorded using a spectrophotometer (UV-2600, Biotechnology Model Services) at wavelengths 666, 653, and 470 nm [34].

### 2.7. Determination of Total Soluble Sugars (TSS)

The TSS was determined by taking 50 mg of fresh leaves. The leaves were immersed in 3 mL of 90% ethanol and incubated (Thermo Stable IS- 20R, DAIHAN Scientific Co., Ltd., Seoul, Republic of Korea) for 1 h at 60–80 °C in a preheated oven. After incubation, the supernatant was extracted and transferred to a fresh sterile tube, and the leftover leaves were crushed in 3 mL of 90% methanol and kept at 80 °C again for 1 h. Later, both supernatants were mixed in a tube with 15 mL of distilled water. A 5% phenolic solution was prepared by dissolving 5 mg of phenolic acid in 100 mL of water. In addition, 1 mL of the sample was extracted from the 5% phenolic solution and mixed with 1 mL of leaf extract. To obtain the final volume, 5 mL of pure sulfuric acid was carefully added to the mixture, followed by the addition of 10 mL of distilled water. The mixture was stirred continuously with a glassware shaker for half an hour before being incubated at 25 °C. At 485 nm, sample absorption was evaluated, and distilled water was employed as a blank solvent [35].

### 2.8. Total Carbohydrates and Fiber Analysis

Next, to analyze total carbohydrates and fiber, 100 mg leaf samples were put into test tubes and 5 mL of HCl (2.5 N) was added [36]. In order to hydrolyze the plant material, the test tubes were immersed in a water bath and heated to boiling temperatures for a total of three hours. Later, the tubes were kept at room temperature to let them cool. To prevent the formation of bubbles, sodium carbonate was added to the solution and mixed in until the desired concentration was reached. Up to 100 mL of distilled water was added to dilute the solution and centrifuged (D2012 plus, DLAB) at 3500 rpm for 10 min. A glucose standard (0.1) was made. Into each test tube, 0.2, 0.4, 0.6, 0.8, and 1 mL solutions were poured. Then, 1 mL of phenolic acid (5%) and 5 mL of sulphuric acid (96%) were added to the mixture, and left at room temperature for 10 min. The test tubes were stirred while they were kept in the water bath at a temperature between 25 and 30 °C for 20 min. After that, the absorbance value was recorded at 490 nm (UV-2600, Biotechnology Model Services).

### 2.9. Measurement of Superoxide Dismutase (SOD) Activity

To calculate the level of SOD, 250 mL of the total reactive homogenous mixture (Nitro blue tetrazolium chloride 15.5 mg, methionine 485 mg, EDTA 10 mg, and Riboflavin 0.02 mg) was employed. The reaction sample was prepared, covered in aluminum foil, and stored in the fridge to avoid deterioration. To measure SOD, 2.725 mL of a homogenous mixture, 25 mL of enzyme extract, and 25 mL of hydrogen peroxide were used. A blank sample (without enzyme extract) was subjected to light (4000 lux for 20 min). Meanwhile, 25 mL of water was utilized as a control specimen. To avoid light, the tubes in the mixture were enveloped in black cloth. The samples’ absorbance was evaluated at 560 nm in glass cuvettes (UV-2600, Biotechnology Model Services) [37].

### 2.10. Measurement of Peroxidase (POD) and Ascorbate Peroxidase (APX) Activity

The activities of POD and APX were evaluated by using the methods reported earlier [38]. In this process, 100 mL of enzyme solution, 1.5 percent guaiacol, and H_2_O_2_ were used in the reaction mixture. The treated samples contained guaiacol (0.1 mL), H_2_O_2_, and potassium phosphate buffer (2.8 mL each). Using a spectrometer (UV-2600, Biotechnology Model Services) with an absorption wavelength of 470 nm, the POD activity was estimated. The buffer was created using potassium phosphate, EDTA-Na2, H_2_O_2_, ascorbic acid, and an enzyme solution. The absorbance was measured at 290 nm.

### 2.11. Quantification of Catalase (CAT) Activity

To analyze the catalase activity, the methods adopted by Aebi were followed [39]. The mixture used in the reaction consists of enzyme extract, potassium phosphate buffer, EDTA-Na_2_ salt, and hydrogen peroxide. At a wavelength of 240 nm, the absorbance was measured (UV-2600, Biotechnology Model Services).

### 2.12. Statistical Analysis of Data

One-way analysis of variance was used (ANOVA). At a threshold of significance of 5%, the least significant difference (LSD) test was performed to differentiate between the means of three replicates that were employed for each treatment.

## 3. Results

### 3.1. PDS Reduces the Inhibitory Effects of TIBA on the Germination of Maize

**Figure 1:**
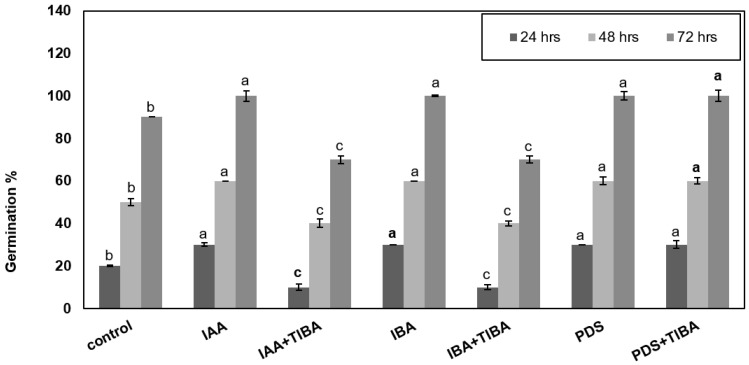
Effect of IAA, IBA, TIBA, and PDS solution on the seed germination of maize. Maize seeds were subjected to control, IAA, IAA + TIBA, IBA, IBA + TIBA, PDS, and PDS + TIBA. Germination was observed after 24, 48, and 72 h. IAA, IBA, PDS, and PDS + TIBA significantly enhance seed germination X-axis represents various treatments. All the data were analyzed by using one-way ANOVA, with multiple comparisons using the LSD test at a significance threshold of *p ≤* 0.05 (*n* = 10). Different letters on graph bars indicate significant differences among different treatments. Each independent experiment was repeated three times.

Maize seeds were treated exogenously with different treatments, i.e., IAA, IBA, and PDS alone and in combination with TIBA, while seeds were also treated with distilled water as a control treatment. The results show that control seeds displayed 20% germination after 24 h, 50% after 48 h, and 90% after 72 h. Seeds treated with IAA, IBA, and PDS alone showed 60% germination after 48 h and 100% after 72 h. Importantly, the application of IAA + TIBA caused a noticeable reduction in the germination rate, being 10% after 24 h, 48% after 48 h, and 70% after 72 h. Similarly, treatment with IBA + TIBA resulted in a germination rate of 8% after 24 h 45% after 48 h and 70% after 72 h. Seeds treated with PDS + TIBA displayed a remarkable increase in germination as compared to IAA + TIBA and IBA + TIBA, while the increase percentage was similar to that for the individual application of PDS, IAA and IBA (Figure 1).

### 3.2. PDS Promotes Plant Height, Leaf Length and Seedling Length under TIBA Treatment

**Figure 2:**
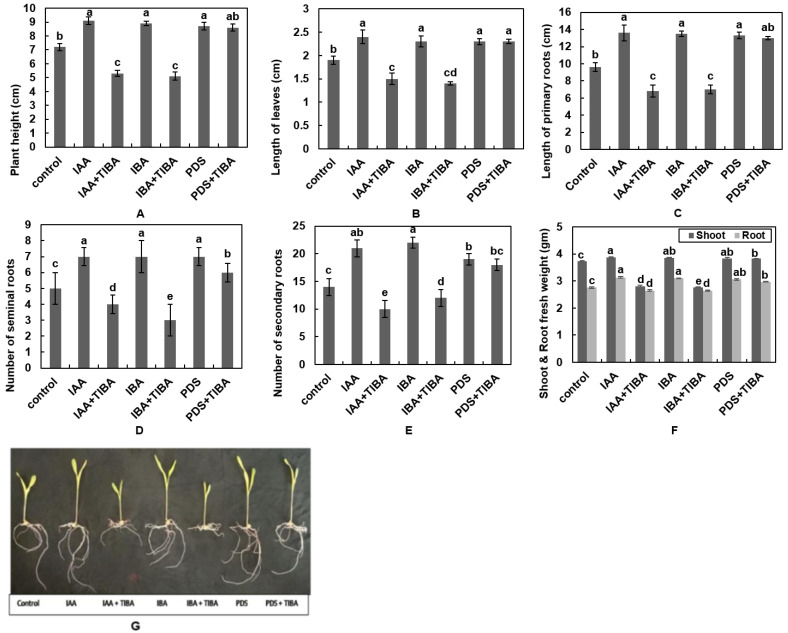
Effect of IAA, IBA, TIBA, and PDS solution on plant height and length of leaves. (**A**) Maize seeds were subjected to the control, IAA, IAA + TIBA, IBA, IBA + TIBA, PDS, and PDS + TIBA. Plant height and length of leaves were observed after 10 days. IAA, IBA, PDS, and PDS + TIBA significantly enhance plant height, (**B**) the length of leaves, (**C**) the length of primary roots, (**D**) the number of seminal roots, (**E**) the number of secondary roots, and (**F**) shoot and root fresh weight, and (**G**) a photograph was taken after 10 days. The X-axis represents various treatments. All of the data were analyzed by using one-way ANOVA, with multiple comparisons using the LSD test at a significance threshold of *p ≤* 0.05 (*n* = 10). Different letters on graph bars indicate significant differences among different treatments. Each independent experiment was repeated three times.

To establish how PDS affects maize morphology in the presence of TIBA, individual and combined treatments of IAA, IBA, PDS with TIBA were applied. The results show increased plant height, i.e., 9.1, 8.9, and 8.7 cm, with the application of IAA, IBA, and PDS, respectively, as compared to the control, for which the plant height was 7.2 cm. However, maize seedling height with the application of IAA + TIBA, IBA + TIBA, and PDS + TIBA was 5.3 cm, 5.1 cm and 8.6 cm, respectively (Figure 2A). These results indicate that the application of TIBA significantly reduces the promoting effects of IAA and IBA on maize height but does not alter the effects of PDS on maize.

Leaves are responsible for the process of photosynthesis, due to which they are known as the food factory of plants. The length of maize leaves was noted as being 1.9 cm in the control, 2.4 cm in IAA, 2.3 cm in IBA, and 2.3 cm under PDS treatment. Contrarily, the responses of maize leaves to IAA + TIBA and IBA + TIBA were found to be the opposite as compared to PDS + TIBA, i.e., 1.5 cm, 1.4 cm and 2.3 cm, respectively (Figure 2B). These results reveal that the application of TIBA did not affect the promoting effect of PDS.

### 3.3. PDS Enhances the Length of Primary Roots, the Number of Seminal and Secondary Roots and the Fresh Weight of Shoots and Roots under TIBA Application

To understand how PDS affects root architecture and biomass under TIBA treatment, maize seedlings were subjected to all individual and combined treatments as mentioned above in the text. The length of the primary roots treated with IAA, IBA, and PDS was 13.3 cm, 13.5 cm, and 13.2 cm, respectively, while the control treatments provided a root length of 9.6 cm, which demonstrates a significant difference in primary root length (Figure 2C,G). On the other hand, treatment with TIBA reduced the promoting effects of IAA and IBA, with primary root lengths of 6.8 and 7 cm, respectively, while PDS remained unaffected, with a 13 cm root length (Figure 2C). Based on these findings, it appears that PDS has the potential to counteract the inhibiting effect of TIBA on the growth of the primary root in maize. However, it is worth establishing how PDS alleviates the inhibitory effects of TIBA.

Seminal roots help plants to absorb water and minerals from the soil to fulfill the basic requirements of plants. In the present study, five seminal roots were observed in the control, while seven were present in the IAA, IBA, and PDS treatments. TIBA treatment alongside IAA, IBA, and PDS reduced the number of seminal roots to four, three, and six, respectively (Figure 2D). The results confer that TIBA reduces the potential of IAA and IBA in seminal roots, while PDS counteracts the inhibiting effect of TIBA on the number of seminal roots.

The effect of PDS on secondary roots under TIBA application was a promoting one, and 18 secondary roots were produced, which represents the maximum number of secondary roots as compared to in IAA + TIBA (10) and IBA + TIBA (12). Seedlings treated with IBA produced 22 and 21 secondary roots in IAA, and 19 were observed in the PDS-treated seedlings (Figure 2E). These results conclude that PDS significantly reduced the inhibitory effect of TIBA on the number of secondary roots.

Shoot and root fresh weights of maize seedlings were determined separately in each experiment. The results show that shoot and root fresh weights were 3.87, 3.13 and 3.85 g under IAA, IBA and PDS treatment. The control displayed 3.74 and 2.76 g of fresh weight. Notably, a remarkable decrease was noticed in the fresh shoot and root weight of maize seedlings treated with TIBA along, with IAA and IBA, at 2.81, 2.65, 2.77, and 2.64 g, respectively, while PDS alleviated the inhibitory effect of TIBA and significantly increased the fresh weight of shoots and roots, which was 3.83 and 2.97 g, respectively (Figure 2F).

### 3.4. TIBA Lowered the Chlorophyll Content in Maize Seedlings under IAA, IBA, and PDS

**Figure 3:**
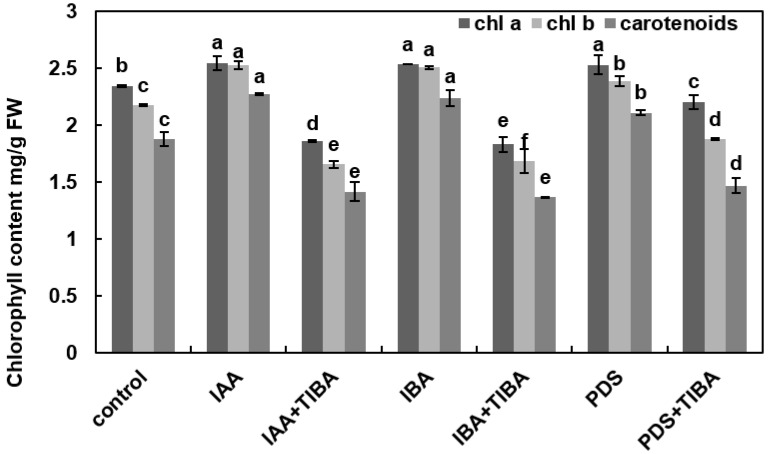
Effect of IAA, IBA, TIBA, and PDS solution on chlorophyll pigments and carotenoid contents of maize. Chlorophyll contents and carotenoid contents were observed on day 10 after cultivation. The chlorophyll and carotenoids contents were measured by randomly selecting 3 plants from each treatment. The *x*-axis represents different treatments. All of the data were analyzed by using one-way ANOVA, with multiple comparisons using the LSD test at a significance threshold of *p ≤* 0.05 (*n* = 3). Different letters on graph bars indicate significant differences among different treatments. Each independent experiment was repeated three times.

Photosynthetic pigments are essential components of photosynthesis in plants. Fresh leaves of 10-day-old maize seedlings were used for the analysis of chlorophyll contents. The results indicated that the level of chlorophyll *a* and *b* in maize seedlings was found to be 2.54 (chlorophyll *a*) and 2.52 mg/g FW (chlorophyll *b*) for IAA, 2.53 (chlorophyll *a*) and 2.50 mg/g FW (chlorophyll *b*) for IBA, and 2.52 (chlorophyll a) and 2.38 mg/g FW (chlorophyll *b*) for PDS, which shows the increase in chl a and b content as compared to the control, which was it was recorded to be 2.34 (chlorophyll *a*) and 2.17 mg/g FW (chlorophyll *b*). Next, TIBA was applied along with IAA, IBA, and PDS. The results show decreased chl *a* and *b* content, i.e., 1.85 mg/g FW and 1.65 mg/g FW with the application of IAA + TIBA and 1.82 mg/g FW and 1.62 mg/g FW with IBA + TIBA, respectively. However, the chlorophyll *a* and *b* content was not altered, i.e., 2.19 mg/g FW and 1.87 mg/g FW, respectively, with the application of PDS + TIBA. These results indicate that PDS has the potential to decrease the inhibitory effects of TIBA on chlorophyll *a* and *b* contents in maize. Next, the carotenoid content was determined in the fresh leaves of maize. The results show that the highest levels of carotenoid content were present in IAA (2.26 mg/g FW) and PDS (2.10 mg/g FW) treated seedlings, while it was (1.87 mg/g FW) in the control. The level of carotenoid content in PDS-treated seedlings was higher. The carotenoid content was decreased by the application of TIBA and IAA, reaching 1.41 mg/g FW; with the application of IBA + TIBA, the level reduced to 1.36 mg/g FW, and with the application of PDS + TIBA, it reduced to 1.46 mg/g FW (Figure 3). These results show that the level of carotenoids was remarkably lowered by the application of TIBA; however, their level was dropped less with the application of PDS + TIBA compared to IAA + TIBA and IBA + TIBA in maize seedlings.

### 3.5. PDS Enhances TSS, Carbohydrates and Fibre Contents in Maize Seedlings under TIBA

**Figure 4:**
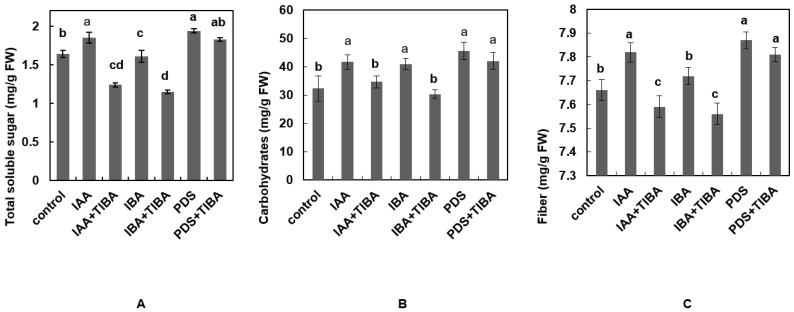
Effect of IAA, IBA, TIBA, and PDS solution on total soluble proteins and total soluble sugars of maize. (**A**) Total soluble sugars were observed and measured by randomly selecting 5 plants from each treatment. (**B**) Carbohydrate and (**C**) fiber contents were observed and measured by selecting 10 plants randomly from each treatment. All of the data were analyzed by using one-way ANOVA, with multiple comparisons using the LSD test at a significance threshold of *p ≤* 0.05 (*n* = 10). Different letters on graph bars indicate significant differences among different treatments. Each independent experiment was repeated three times.

Sugars are primary metabolites that are essential for the growth and development of plants. To investigate the levels of TSS under IAA, IBA and PDS alone and combined with TIBA, experiments were carried out. The results indicate that the level of TSS was increased by IAA and PDS, at 1.85 and 1.94 mg/g FW, respectively, while in the control, it was 1.64 mg/g FW. Noticeably, the level of TSS in IBA-treated seedlings was 1.61 mg/g FW, which is less than that in the control seedlings. The combined application of IAA and IBA with TIBA decreased TSS content to 1.24 and 1.15 mg/g FW, respectively, while the TSS content in PDS + TIBA remained unaltered (1.94 mg/g FW) (Figure 4A). The above results demonstrate that PDS counteracts the application of TIBA and did not affect the TSS concentration.

Next, total carbohydrate content was analyzed in maize under different treatments. The result shows that the maximum level of carbohydrates was present in seedlings treated with PDS, at 45.6 mg/g FW, followed by IAA and IBA, at 41 mg/g FW and 41.66 mg/g FW, respectively. The content of carbohydrates in the control was 32.33 mg/g FW. It was also noted that the application of TIBA along with IAA and IBA reduced the content of carbohydrates. The content of carbohydrates in IAA + TIBA was 34.66 mg/g FW, and 30.33 mg/g FW in IBA + TIBA. The carbohydrate content quantified in PDS + TIBA was 42 mg/g FW (Figure 4B).

Fiber analysis was also performed in maize seedlings under different treatments. The results indicate that seedlings treated each with PDS and IAA resulted in a maximum level of fiber content, at 7.87 and 7.82 mg/g FW, respectively. This was remarkably comparable with the control and IBA-treated seedlings, which provided values of 7.66 and 7.72 mg/g FW. The fiber content in IAA + TIBA was 7.59 mg/g FW and in IBA + TIBA it was 7.56 mg/g FW. In PDS + TIBA, the fiber content was 7.81 mg/g FW, which shows that fiber content remained unaffected (Figure 4C).

### 3.6. PDS Decreased the Levels of SOD, POD, APX and CAT Activity under TIBA Stress

**Figure 5:**
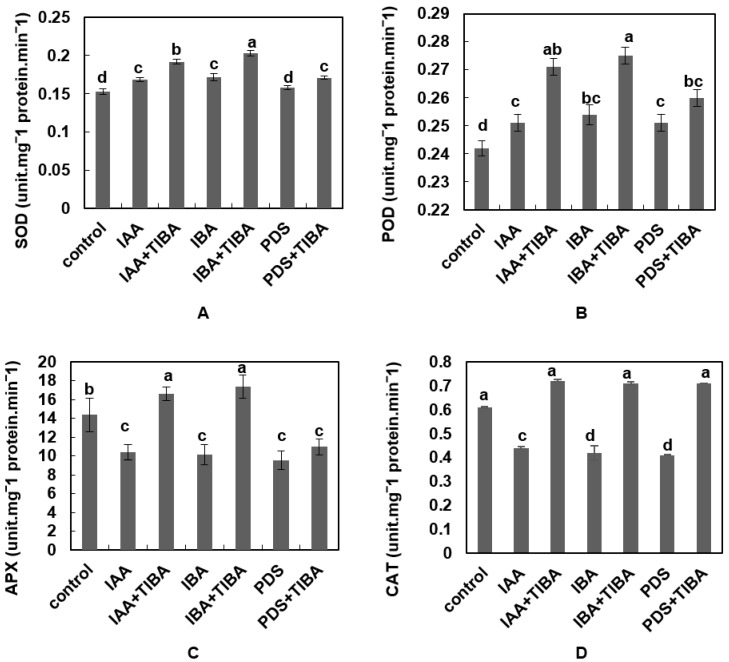
Effect of IAA, IBA, TIBA, and PDS solution on SOD, POD, APX, and CAT activity in maize. (**A**) SOD, (**B**) POD, (**C**) APX, and (**D**) CAT activities were observed and measured by randomly selecting 10 plants from each treatment. All of the data were analyzed by using one-way ANOVA, with multiple comparisons using the LSD test at a significance threshold of *p ≤* 0.05 (*n* = 10). Different letters on graph bars indicate significant differences among different treatments. Each independent experiment was repeated three times.

The level of SOD was found to be decreased under PDS and IAA treatments. The level of SOD under IAA, IBA and PDS treatment was 0.169, 0.172, and 0.158 unit.mg^−1^ protein.min^−1^, respectively, while in the control it was 0.153 unit.mg^−1^ protein.min^−1^. Next, the level of SOD was determined under TIBA along with IAA, IBA, and PDS treatments. The results show that the SOD level increased in seedlings treated with IAA + TIBA, reaching 0.192 unit.mg^−1^ protein.min^−1^, while in those treated with IBA + TIBA, the SOD content was 0.203 unit.mg^−1^ protein.min^−1^. The level of SOD was measured as being 0.171 unit.mg^−1^ protein.min^−1^ in seedlings subjected to PDS + TIBA (Figure 5A).

The activity of POD was low (0.251, 0.251 and 0.254 unit.mg^−1^ protein.min^−1^) in IAA-, IBA-, and PDS-treated maize seedlings, respectively. The POD value in the control was 0.242 unit.mg^−1^ protein.min^−1^. Meanwhile, TIBA increased the POD level in maize seedlings, i.e., with IBA + TIBA, the level was 0.275, with IAA + TIBA, the level was 0.271 unit.mg^−1^ protein.min^−1^, and with PDS + TIBA, the value was 0.260 unit.mg^−1^ protein.min^−1^ (Figure 5B).

The content of APX was determined in maize seedlings treated with different treatments. The results show that the highest level of APX activity was found in IBA + TIBA and IAA + TIBA, at 17.37 and 16.64 (unit.mg^−1^ protein.min^−1^), respectively, while in seedlings treated only with IBA and IAA, the level of APX was 10.16 and 10.44 unit.mg^−1^ protein.min^−1^, respectively. Similarly, maize seedlings treated with PDS alone and in combination with TIBA exhibited a low level of APX, and the content was 9.55 and 10.99 unit.mg^−1^ protein.min^−1^, respectively. The control resulted in an APX activity of 14.36 unit.mg^−1^ protein.min^−1^ (Figure 5C). These results show that PDS can significantly alleviate the adverse effect of TIBA by reducing the content of APX as compared to IAA and IBA.

The activity of CAT was decreased in seedlings treated alone with IAA, IBA, and PDS, i.e., 0.44, 0.42, and 0.41 (unit.mg^−1^ protein.min^−1^), respectively. In the control, the level of CAT was higher (0.61 unit.mg^−1^ protein.min^−1^) than in seedlings treated with IAA, IBA, and PDS. The content of CAT in seedlings treated with IAA + TIBA was 0.072 unit.mg^−1^ protein.min^−1^, while in those treated with IBA + TIBA and PDS + TIBA, it was 0.71 unit.mg^−1^ protein.min^−1^ (Figure 5D).

## 4. Discussion

The success or failure of a crop production cycle often depends on the rate and success of seed germination [40]. In the current study, we examined seed germination, plant height, leaf length, root architecture, chlorophyll content, antioxidant activity, TSS, and carbohydrate and fiber contents under various treatments including the control, IAA, IBA, IAA + TIBA, IBA + TIBA, PDS and PDS + TIBA. The results show that PDS accelerated seed germination under TIBA treatment, whereas TIBA deceased the germination rate when seeds were treated with either IAA or IBA. Similarly, plant height, leaf length, and number of primary, seminal roots and lateral roots were found to improve when the seedlings were subjected to either individual or combined treatment with PDS and TIBA.

Chlorophyll content, TSS, and carbohydrate and fiber contents were enhanced by PDS, IAA and IBA treatment. Similarly, the combined treatment with PDS and TIBA increased their levels, while IAA and IBA treatment with TIBA reduced their levels. Importantly, the antioxidative level of the maize was modulated by PDS under TIBA treatment.

A previous study showed that karrikins accelerate the seed germination process by mediating the signaling transduction of various plant hormones such as ABA, auxin, and gibberellin [41]. Indole-3-acetic acid is involved in enhancing the seed germination process of several plant species, such as tobacco, Brazilian pine, and sunflower [42]. Auxin (IAA and IBA) acts as a powerful regulator of cell growth, cell division, and cell differentiation [43]. IAA is a phytohormone that controls a wide variety of the developmental and morphological processes taking place in plants. Previous studies showed the possible mode of action of karrikins that accelerates seed germination. During seed germination, karrikins inhibit the biosynthesis of ABA, which causes seed dormancy and enhances the biosynthesis of GA. The biosynthesis of ABA is inhibited by suppressing the ABA2 gene, which induces the pathway of ABA synthesis and upregulates ABI3, resulting in the inhibition of seed germination. On the other hand, karrikins regulate GA biosynthesis by inducing the expression of the GA3x gene, which regulates seed germination [44,45,46]. Looking at the mode of action of karrikins and their effect on phytohormones during seed germination, it is likely that PDS might regulate seed germination by maintaining a balance between GA and ABA synthesis. PDS may biosynthesize GA via inducing the expression of GA3x, which further enhances seed germination. On the contrary, PDS inhibits the synthesis of ABA by suppressing ABA-responsive genes, i.e., ABA2 and ABI3, which results in seed germination. The above results demonstrate that PDS may play a regulatory role in the phytohormone signaling pathway.

Recent reports have also shown that PDS solutions are involved in the acceleration of seed germination along with root/shoot length in different plants [47,48,49]. Accumulating evidence suggests that it is an accelerator of seed germination in many different species, such as *Gyrostemon ramulosus*, *Gyrostemon recemiger*, *Anigozanthos flavidus*, *Zea mays,* and also *Apium graveolens* [50,51,52]. It was also previously reported that PDS is involved in enhancing the growth of roots and also accelerating the shoot growth of several plants, such as okra, bean, tomato, and rice [52,53,54]. An active class of chemicals in PDS called butenolides, also known as karrikins, are involved in accelerating the germination process and plant growth [55]. Butenoloid has been reported to enhance callus growth when applied alone or in combination with kinetine in soya bean and to increase root growth in mung bean when supplemented with IBA. The mode of action of this active butenolide compound is still unknown. However, it has been suggested that smoke compounds act either by modulating the sensitivity of the tissue to PGRs, activating enzymes or by modifying the receptor molecules [56]. These findings suggest that PDS may follow the same mode of action as butenoloid does, supporting our results that IAA, IBA, and PDS alone enhanced the germination of maize seeds, and also that PDS significantly alleviates the inhibitory effect of TIBA on the germination of seeds.

Previous studies showed that N-1-naphtylphtalamic acid (NPA) and 2,3,5-triiodobenzoic acid (TIBA) inhibited auxin transport in plants [57]. Recent research has shown that auxin transport inhibitors like TIBA can bind to the regulatory region of an auxin efflux carrier complex and effectively impede auxin transport [58]. Our results also show that IAA and IBA with TIBA significantly accelerate seedling growth, including the number of roots and leaves, the length of roots and shoots, and the fresh weight of roots and shoots in maize. Altogether, our findings, along with other findings, confirm that exposure to TIBA decreases the activity of IAA and IBA, but does not affect the positive effects of PDS solution on the seed germination and seedling growth of maize.

The plant’s roots are its most fundamental and important component, as they are directly responsible for the development of the plant. Auxin plays a role in a variety of activities that take place throughout plant development, including root growth [59,60]. The term “auxin” refers to a group of naturally occurring compounds (IAA and IBA) that have the ability to control certain aspects of plant growth. These aspects include the curvature of the *Avena coleoptile* tips, the length of hypocotyls and epinastic leaves, the initiation of lateral and adventitious roots, and the growth of primary roots and the cotyledons [43,61,62]. IAA controls the growth of both the primary and lateral roots in plants [63,64,65,66].

A previous research work reported that the length of adventitious roots, the number of adventitious roots, and also the length of lateral roots in sweet potatoes are enhanced by the application of PDS [67]. Our results show that the number of primary, seminal and lateral roots, root fresh weight, and the length of primary roots were significantly enhanced by the application of IAA, IBA, PDS, and PDS + TIBA in maize plants. Our results also show that the application of TIBA negatively affects the activity of IAA and IBA. However, TIBA did not alter the promoting effects of the PDS solution.

Photosynthetic pigments are the most essential components for the process of photosynthesis in plants. Proteins and sugars are also very important primary metabolites that play a significant role in plant growth. Our results show that photosynthetic pigments like chlorophyll a and b along with carotenoid contents and total soluble sugars are increased by IAA, IBA, PDS, and PDS + TIBA in maize plants. Our results also show that the application of TIBA with IAA and IBA decreased the photosynthetic pigments and total soluble sugars in maize but did not alter the positive effects of the PDS solution in maize. Previously, it was reported that IAA and IBA are involved in enhancing the chlorophyll and carotenoid contents in chickpeas, and guava [68]. IBA also has positive effects on protein contents in *Tectonagrandis* L. [69]. 2, 3, 5-Triiodo benzoic acid has been found to reduce the photosynthetic pigments in *Oryza sativa* [70]. Conversely, IAA has been reported to increase the total soluble proteins and total soluble sugars in soybean [71].

PDS has been reported to elevate the level of total soluble sugar starch, protein content, lipase activity, and lipid content in lettuce [72,73]. Our results are parallel with previous research studies which showed that PDS could be a promoting factor to enhance the growth and biochemical parameters of plants. Antioxidative enzymes constitute the defense system of plants. According to our results, IAA, IBA and PDS modulate the levels of antioxidant enzymes including SOD, POD, APX and CAT.

IAA has been reported to play a vital role in the production of antioxidant enzymes under stress conditions [74]. It has also been reported that the application of IAA enhances the production of antioxidant enzymes such as SOD, POD and CAT in plants, which help in the scavenging of ROS produced in plants [74,75,76]. Recent studies showed that PDS reduces the level of antioxidants in plants under stress conditions [77,78]. These studies are in line with our results, which show that PDS has the capability to decrease enzymatic activities under TIBA stress.

## 5. Conclusions and Future Recommendations

The current research proved that IAA, IBA, and PDS have the potential to enhance the morphological and biochemical parameters of maize including seed germination, seedling growth, and photosynthetic pigments, especially chlorophyll *a* and *b*, carotenoids, and total soluble sugars. Our findings confirm that TIBA significantly inhibits the role of IAA and IBA in maize growth. However, TIBA does not affect the positive effects of PDS on maize morphological and biochemical growth parameters. It can be deduced that PDS either competes with TIBA for auxin channels and blocks the entry of TIBA or it increases the level/concentration of internal auxin, which might be sufficient for it to compete with TIBA for auxin carrier channels; however, establishing how PDS achieve this requires further work. Moreover, the quantification of endogenous auxin under PDS as well as PDS + TIBA may help in finding the reason for why PDS activity is not influenced by TIBA. Further work is required to extend this work by investigating the effects of PDS and TIBA on the reproductive stage of maize to see their effect on maize seed production. To confirm, whether PDS acts like a hormone need further research. The mitigating effects of PDS on TIBA need to be investigated in other plant species. Studies are also required to analyze the mechanism of action of PDS and how it alleviates the phytotoxic effect of TIBA.

## Data Availability

All related data are within the manuscript.
